# Two-Year Follow-Up of a Multi-centre Randomized Controlled Trial to Study Effectiveness of a Hospital-Based Work Support Intervention for Cancer Patients

**DOI:** 10.1007/s10926-019-09831-8

**Published:** 2019-02-16

**Authors:** S. J. Tamminga, J. H. A. M. Verbeek, M. M. E. M. Bos, G. Fons, J. J. E. M. Kitzen, P. W. Plaisier, M. H. W. Frings-Dresen, A. G. E. M. de Boer

**Affiliations:** 1 Coronel Institute of Occupational Health, Amsterdam Public Health Research Institute, Amsterdam UMC, University of Amsterdam, Meibergdreef 9, Amsterdam, The Netherlands; 2 Finnish Institute of Occupational Health, Kuopio, Finland; 3 Department of Internal Medicine, Reinier de Graaf Groep, Delft, The Netherlands; 4 Department of Gynaecology, Academic Medical Center, University of Amsterdam, Amsterdam, The Netherlands; 5 Department of Internal Medicine, Albert Schweitzer Hospital, Dordrecht, The Netherlands; 6 Department of Surgery, Albert Schweitzer Hospital, Dordrecht, The Netherlands

**Keywords:** Neoplasms, Return to work, Oncology service, Hospital, Program effectiveness, Randomized controlled trial

## Abstract

**Abstract:**

*Purpose* Purpose is to: (1) study effectiveness of the hospital-based work support intervention for cancer patients at two years of follow-up compared to usual care and (2) identify which early factors predict time to return-to-work (RTW). *Methods* In this multi-center randomised controlled trial (RCT), 106 (self-)employed cancer patients were randomized to an intervention group or control group and provided 2 years of follow-up data. The intervention group received patient education and work-related support at the hospital. Primary outcome was RTW (rate and time) and quality of life (SF-36), and secondary outcomes were, work ability (WAI), and work functioning (WLQ). Univariate Cox regression analyses were performed to study which early factors predict time to full RTW. *Results* Participants were diagnosed with breast (61%), gynaecological cancer (35%), or other type of cancer (4%). RTW rates were 84% and 90% for intervention versus control group. They were high compared to national register-based studies. No differences between groups were found on any of the outcomes. Receiving chemotherapy (HR = 2.43, 95% CI 1.59–3.73 p < 0.001), low level of education (HR = 1.65, 95% CI 1.076–2.52 p = 0.02) and low work ability (HR = 1.09 [95% CI 1.04–1.17] p = 0.02) were associated with longer time to full RTW. *Conclusions* We found high RTW rates compared to national register-based studies and we found no differences between groups. Future studies should therefore focus on reaching the group at risk, which consist of patients who receive chemotherapy, have a low level of education and have a low work ability at diagnosis.

**Trial registration:**

Netherlands Trial Registry (NTR) (http://www.trialregister.nl/trialreg/admin/rctview.asp?TC=1658): NTR1658.

## Introduction

Due to improvement in survival rates, returning to paid work after a cancer diagnosis is of increasing relevance to cancer patients, their families, employers, and the society at large [1]. However, cancer patients have a 1.4 higher chance of becoming unemployed compared to ‘healthy’ individuals [2]. Besides, when able to return-to-work (RTW), many cancer patients have to make involuntary work changes such as working less hours [3] or experience fewer possibilities for development [4]. This is unfortunate, since work is important to cancer patients. That is, because work provides self-esteem, a return to normalcy, a better quality of life, and a better financial situation despite income protection provided by the social security system [5–7].

Reasons for not being able to RTW are not primarily medical. It is often an interaction between the person (e.g., person- and disease related factors) and the environment (e.g., work-related factors and culture and social security system context) [8–12].

Since there is ample room to improve the RTW of cancer patients, we developed a hospital-based work support intervention for cancer patients. This was based on a systematic literature review on work-related interventions for cancer patients [13] and interviews with cancer patients [14] and relevant stakeholders [15]. To be able to deliver an early intervention, the intervention is delivered at the hospital as part of psycho-oncological care. We chose this time frame deliberately, because the longer the duration of sick-leave the more difficult it is to RTW [16]. Furthermore, most cancer patients do not have contact with their employer or occupational physician early in the treatment process [17].

The core elements of the intervention are to address RTW structurally as work is not typically addressed at the hospital, and to alter self-assessed work ability positively with patient education and support. We decided on these elements as work ability has been identified as one of the main amenable prognostic factors of RTW [9].

To be able to study effectiveness of the hospital-based work support intervention, we developed a multi-centre randomised controlled trial with a follow-up of 2 years [15]. Results of this study at 1 year of follow-up showed high RTW rates in both the intervention group and the control group, which did not differ between groups [18]. Additionally, the process evaluation of this study showed that the intervention was highly appreciated by patients and was easy to implement in psycho-oncological care [19]. To be able to conclude whether the intervention has an effect at the long-term follow-up, we studied the results of 2-years of follow-up separately. This is in our opinion of added value to the literature in which little longitudinal study results of RCTs on RTW for cancer patients have been reported. First, since the duration of cancer treatment and rehabilitation after oncological rehabilitation can take easily more than 1 year [20], we assume that significant changes in the RTW process might occur between 1 and 2 years of follow-up. Second, employees with a permanent position are protected against discharge due to sickness absence during the first 2 years of sick leave. Both the employer and the employee have rights and obligations regarding RTW as laid down in the Dutch law. Therefore, it might be that part of the intervention i.e. drawing up a concrete and gradual RTW plan, might have an effect after 1 year of sickness absence.

In addition to studying effectiveness of the hospital-based support intervention on RTW (either part-time or full-time), studying which factors predict time to RTW at 2 years of follow-up could provide us with additional understanding which factors predict time to RTW. Non-amendable factors provide us insight, which are the characteristic of the group at risk, which group should thus receive a more intense intervention [21].

The aim of this study is twofold: (1) to study effectiveness of the hospital-based work support intervention for cancer patients at 2 years of follow-up and (2) to identify which early factors predict time to RTW at 2 years of follow-up.

## Method

The study was approved by the medical ethics committee of the Academic Medical Center [MEC 08/267] and by the local medical ethics committee of each participating hospital. The study was registered at the Dutch National Registry:NTR1658 (http://www.trialregister.nl/trialreg/admin/rctview.asp?TC=1658).

The design of the study and the content of the hospital-based work support intervention have been described in detail elsewhere [15]. We used items from the CONSORT statement for improving the quality of reporting randomised trials [22].

### Patients

The inclusion criteria were: (1) being diagnosed with cancer; (2) between 18 and 60 years of age; (3) treated with curative intent at one of the participating hospital departments. This was defined as an expected 1-year survival rate of approximately 80%; (4) having paid work; and (5) being on sick leave. Patients were excluded if they were not sufficiently able to speak, read, or write Dutch, had a severe mental disorder or other severe co-morbidity, and for whom the primary diagnosis of cancer had been made more than 2 months previously.

Cancer patients who were diagnosed at one of the participating hospital departments between May 2009 and December 2010 and who were eligible and willing to participate were enrolled in the study. The enrolment of new patients ended in December 2010 to enable the inclusion of patient follow-up data within the time constraints of the study.

### Hospital-Based Work Support Intervention

The hospital-based work support intervention started at the onset of the study and was spread across a maximum of 14 months [15]. It consisted of: (1) delivering patient education and support at the hospital by an oncology nurse or medical social worker (hereafter referred to as nurse), integrated into the usual psycho-oncological care in the form of 4 meetings that lasted 15 min each; (2) improving communication between the treating physician and the occupational physician by sending at least one letter to the occupational physician containing information about cancer patient’s diagnosis and treatment. This was only done in case a patient provided his/her consent to allow medical information to be sent from a treating physician to an occupational physician (as is laid down in the Dutch law); and (3) drawing-up a concrete and gradual RTW plan. We asked the occupational physician to organise a meeting between the patient and the employer to draw-up a RTW plan.

### Study Design

This study was a multi-centre randomised controlled trial with a follow-up of 2 years. Six hospitals in the Netherlands participated in the study. Based on the inclusion and exclusion criteria, the treating physician or nurse informed only eligible cancer patients of the study 2–3 weeks after their cancer diagnosis. Those who were also willing to participate were enrolled in the study. After the patients had filled in the baseline questionnaire, one of the authors [ST] performed randomisation to the intervention or to the control group using the computerised randomisation programme ALEA [23]. The allocation ratio was set as equal in the programme. Stratified randomisation was applied for two important prognostic factors of RTW [24]; age (< 50 or ≥ 50) and cancer diagnosis (i.e. hospital department). Minimisation was applied to equalise group sizes. Each consecutive patient was entered in the programme and according to the conditions mentioned above, the programme randomly assigned each patient to the intervention group or to the control group. The allocation was irrevocable and was not changed during the study nor during the analyses. Patients and providers were immediately informed of the allocation, as it was impossible to conceal allocation for this intervention.

Patients were asked to fill in a questionnaire at baseline and at 6, 12, 18, and 24 months of follow-up. The follow-up questionnaires were mailed to the patient’s home address with a postage-paid envelope enclosed.

### Measurements

The primary outcomes were RTW and quality of life. The intervention was considered effective if patients in the intervention group had a statically significant shorter time to RTW (in days) than patients in the control group, provided that their quality of life had not deteriorated statistically significant.

RTW was operationalized as the rate of RTW at 2 years of follow-up. RTW was also operationalized as the number of calendar days between the first day of sick leave and the first day at work (either part-time or full-time) that was sustained for at least 4 weeks. Quality of life was assessed with the Short Form-36 (SF-36) [25]. All subscales were taken into account and a Visual Analogue Scale (VAS). Higher scores on each subscale represent a better quality of life score.

Secondary outcomes were work ability and work functioning. Work ability was measured using the first three questions of the Work Ability Index (WAI) [26], i.e., total, physical and mental work ability [27]. Total work ability was measured on a 0–10 scale and physical and mental work ability on a 5-point Likert Scale. Higher scores means better work ability. Work functioning was measured with the Work Limitation Questionnaire (WLQ) [28]. All subscales were taken into account and a higher score on each subscale represents lower work functioning.

The socio-demographic factors measured at baseline were: number of days between the first day of sick leave and start of the study, marital status, time since diagnosis, breadwinner status, position at work, shift work, years in current position, years of paid employment, income, importance of work (measured on a Visual Analogue Scale (VAS)-scale), and company size.

Prognostic factors of RTW were: age, gender, level of education (low, medium, high), cancer diagnosis, cancer treatment, number of working hours according to contract, physical workload (Questionnaire of Perception and Judgement of Work (VBBA)) [29], fatigue (Multidimensional Fatigue Inventory (MFI)) [30], depression (Centre for Epidemiologic Studies for Depression Scale (CES-D)) [31], co-morbidity, and self-efficacy (general self-efficacy scale (ALCOS)) [32].

### Sample Size

The calculation of the patient sample size was based on two earlier studies focused on RTW in cancer patients [15, 30]. Based on the RTW rates in these studies, we assumed a relative risk of not returning to work of 0.53 for individuals in the intervention group versus those receiving usual care [12]. With a power of 80% and two-sided significance level of p < 0.05, the sample size required was 109 patients in each group [31]. Assuming that 20% of the initial patients would be lost to follow-up, 270 patients should be recruited to gather 246 patients at 2 years of follow-up. To account for at least 10% missing data at baseline, 300 patients sought to be included in the study.

### Statistical Analysis

Data entry was verified by a 100% double data check of the primary outcome RTW. All analyses were performed according to the intention-to-treat principle. However, we censored patients in the Kaplan–Meijer survival analysis who dropped out of the study. Therefore, differences between patients who dropped out or completed the study were analysed according to their baseline quality of life scores. All data were analysed by means of descriptive statics using IBM SPSS version 20 (New York USA). We considered a p-value ≤ 0.05 to be statistically significant.

### Effectiveness of the Hospital-Based Work Support Intervention

We calculated relative risks and 95% confidence interval for returning to work (full and partial) at 2 years of follow-up for the intervention group versus the control group. The median time to RTW was analysed with a Kaplan–Meier survival analysis, and differences between groups was tested with the log rank test. In addition, the Cox proportional hazard model of survival analysis was applied to estimate hazard ratios and the corresponding 95% confidence intervals for the time to full RTW with a hazard ratio > 1 indicating a longer time to RTW.

Improvements in the subsequent primary outcome of quality of life and the secondary outcomes of work ability and work functioning between groups were examined using longitudinal multilevel analyses.

### Predictors of Time to RTW

Univariate Cox regression analyses were performed to identify whether the following variables predict time to RTW.

Socio-demographic variables: (i) age; (ii) marital status (married or living with partner versus not married or living with partner); and (iii) level of education (low/middle vs. high).

Health-related variables: (i) diagnosis (breast cancer versus gynaecological cancer); (ii) cancer treatment (chemotherapy yes/no, radiotherapy yes/no, surgery yes/no, hormone treatment yes/no); (iii) co-morbidity (none vs. one or more); (iv) fatigue (general fatigue measured with MFI); (v) depression (total score CES-D); and (vi) self-efficacy (ALCOS).

Work-related variables: (i) overall work ability (first question of WAI); (ii) work ability physical work load (second question of WAI); (iii) work ability mental work load (third question of WAI); and (iv) type of contract (fixed vs. temporary or self-employed), physical workload (measured with VBBA).

For treatment, we used a time-depended co-variate to only include those who received cancer treatment before the outcome of interest (i.e. RTW) took place. We considered a p-value ≤ 0.05 to be statistically significant.

## Results

**Fig. 1:**
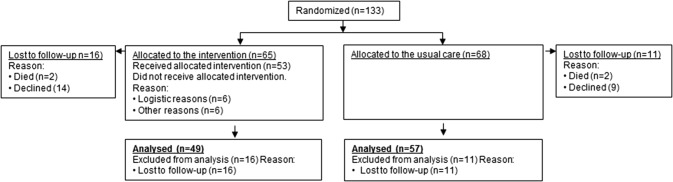
Patient flow

In total, 133 cancer patients were included in the study; 65 were assigned to the intervention group and 68 were assigned to the control group (Fig. 1). In this study only the 106 patients who provided 2 years of follow-up data were included. At 2 years of follow-up, RTW status was reported by 49 patients (75% response) in the intervention group and 57 patients (84% response) in the control group. Reasons for loss to follow-up included, choice to decline (2 patients) or were unknown (21 patients), and 4 patients died within the 2 years of follow-up period. Average age at baseline was 47.5 ± 7.9 years (Table 1). Cancer diagnoses included breast cancer (61%), cervix cancer (23%), cancer of the ovaries (9%) or vulva (4%), and other (3%) (Table 1). The number of co-morbidities at baseline in the intervention group was statistically significant higher compared to the control group (p = 0.045). Patients in the intervention group reported at baseline (before randomisation) statistically significant higher importance of work compared to patients in the control group (p = 0.038). No other differences between groups were found (Table 1).

**Table 1:** Patient characteristics at baseline

Patient characteristics		Intervention group (N = 49)	Control group (N = 57)	p-Value
Socio-demographic characteristics*				
Age (years)		47.1 ± 8.2	47.8 ± 7.6	0.66
Gender (N (%) female)		48 (98%)	57 (100%)	0.28
Marital status (N (%) married or living with partner)		41 (84%)	40 (70%)	0.10
Breadwinner position (N (%) sole or shared)		29 (60%)	21 (37%)	0.12
Education level (N (%))				
Low		3 (6%)	9 (16%)	0.22
Intermediate		30 (61%)	28 (49%)	
High		15 (31%)	20 (35%)	
Clinical characteristics^*^				
Diagnosis (N (%))				
Breast cancer		30 (61%)	35 (61%)	0.33
Cervix cancer		11 (22%)	13 (23%)	
Ovarian cancer		3 (6%)	7 (12%)	
Vulva cancer		2 (4%)	2 (4%)	
Other		3 (6%)	0 (0%)	
Number of co-morbidities (N (%))				0.045^#^
0		25 (52%)	32 (56%)	
1		8 (16%)	17 (30%)	
≥ 2		16 (33%)	8 (14%)	
Surgery (N (%) yes)		41 (84%)	46 (81%)	0.69
Chemotherapy *(N (%) yes)*		30 (61%)	33 (58%)	0.72
Radiotherapy *(N (%) yes)*		25 (51%)	26 (46%)	0.58
Hormone treatment *(N (%) yes)*		18 (37%)	20 (35%)	0.86
Work-related characteristics^*^				
Type of occupation (N (%))				
Health care		11 (22%)	13 (23%)	0.76
Education		8 (16%)	10 (18%)	
Administrative		4 (8%)	6 (11%)	
Sales		3 (6%)	7 (12%)	
Other		23 (47%)	21 (37%)	
Type of work (N (%) mainly physically work)		15 (31%)	22 (39%)	0.39
Physical workload (0–28)^**^		4.6 ± 3.9	5.4 ± 4.0	0.32
Time since sick listed (days)		24.1 ± 35.9	18.1 ± 22.3	0.92
Importance of work (0–100)^**^		60.4 ± 22.6	49.6 ± 28.9	0.038^#^
Shift work (N(%) shift work)		12 (25%)	10 (18%)	0.38
Type of contract (N (%))				0.27
Permanent		46 (93%)	48 (84%)	
Temporary		3 (6%)	5 (9%)	
Self-employed		0 (0%)	2 (4%)	
Other		0(0%)	2 (4%)	
Health-related characteristics^*^				
Fatigue (MFI)^**^	General fatigue (0–20)	12.4 ± 5.0	12.8 ± 4.5	0.30
Depression (CES-D)^**^	Sum score (0–60)	13.9 ± 9.7	12.2 ± 7.1	0.33
Self-efficacy (ALCOS)^**^	Sum score (0–80)	68.0 ± 8.0	67.0 ± 7.5	0.45

Percentages do not always add up due to rounding.

*Continuous variables: mean ± standard deviation; nominal and ordinal variables number and percentages

**Higher scores represent higher level of physical workload, importance of work, fatigue, feelings of depression, and self-efficacy

#p ≤ 0.05

### Hospital-Based Work Support Intervention

Detailed information about use of the hospital-based work support intervention was published elsewhere [19]. In short, 88% of the patients assigned to the intervention group received the patient education and support from the nurse. Eighty-six percent of the patients provided consent to send a letter from the treating physician to the occupational physician. In five cases (10%), the patient’s occupational physician organized a meeting between the patient, supervisor, and himself to draw-up a RTW plan.

### Primary Outcome—RTW and Quality of Life

**Fig. 2:**
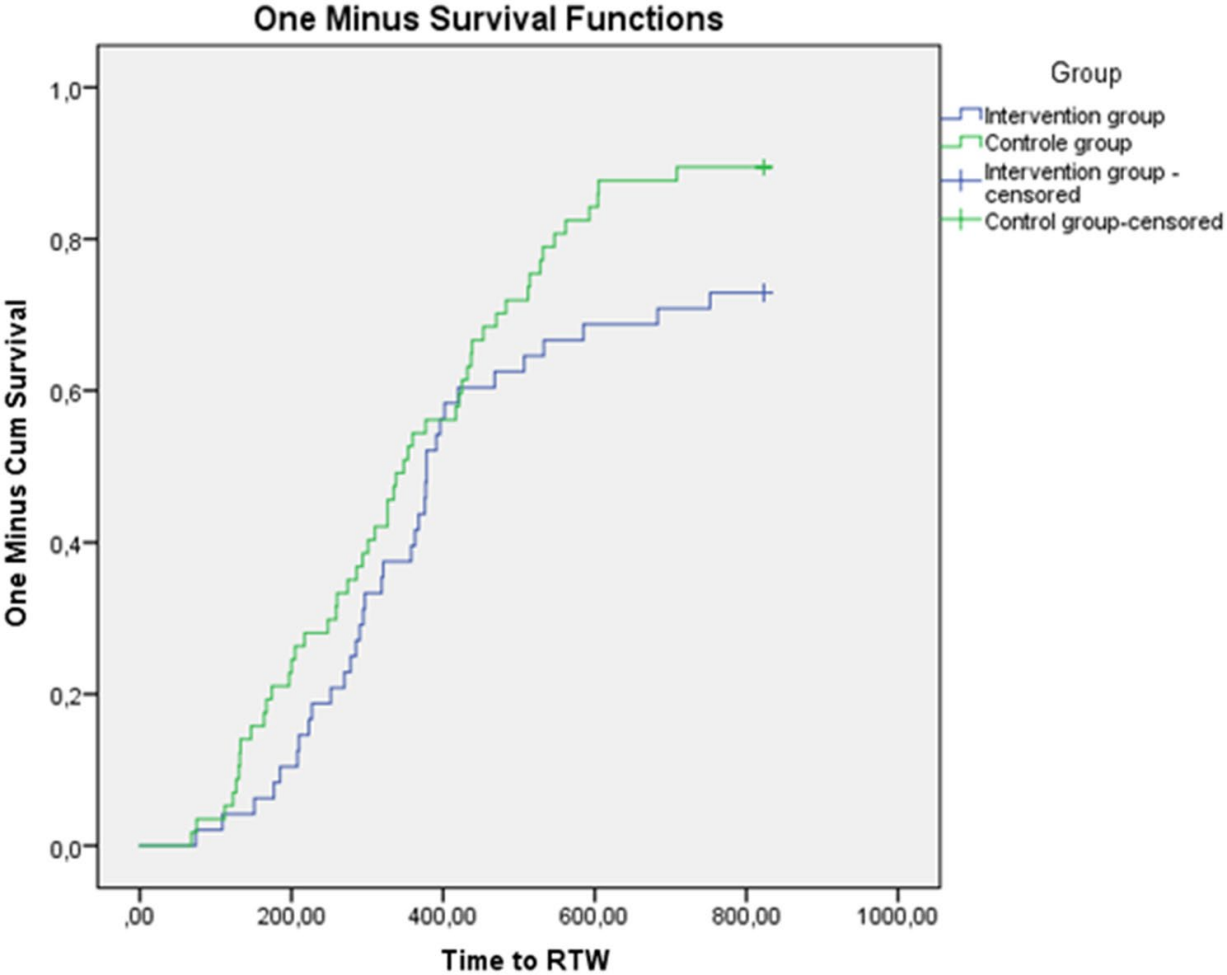
Time to return to work

The RTW rate (either full or partial) of the 106 randomised patients with follow-up data at 2 years of follow-up was 84% for the intervention group and 90% for the control group (p = 0.27). The relative risk of returning to work (either full or partial) for the intervention group versus the control group was 0.60 (95% CI 0.19–1.8). Of the patients who did not RTW (intervention versus control group); 4 versus 3 lost their job, 2 versus 0 applied for or received work disability pension, and 2 versus 3 were again on sick leave. Median time from initial sick leave to full RTW was 363 days (range 19–832) for the intervention group and 344 days (range 136–922) for the control group (log rank test; p = 0.062). Figure 2, summarizes the Kaplan–Meier survival analyses for the two groups for time to full RTW, with the one minus survival function indicating the proportion of patient returning to work over time. Median time from the initial sick leave to partial RTW was 307 days (range 136–922) for the intervention group and 435 days (range 357–768) for the control group (log rank test; p = 0.077).

Quality of life scores showed statistically significant improvements over time (p ranged between ≤ 0.001 and ≤ 0.05) in both groups, especially from baseline to 1 year of follow-up and stated stable or decreased slightly from 1 year of follow-up to 2 years of follow-up. The interaction effect of time and group did not differ between groups (Table 2).

**Table 2:** Quality of life, work ability, and work functioning scores at baseline, 1 year and 2 years of follow-up

	Group	Baseline	1 year follow-up	2 years follow-up	p-Value^**^
Quality of life^*^ (SF-36) (0–100) (N = 81)					
Physical functioning	Intervention	76 ± 29	81 ± 15	83 ± 18	0.82
	Control	74 ± 27	79 ± 19	81 ± 20	
Role-physical	Intervention	48 ± 44	46 ± 41	65 ± 46	0.92
	Control	53 ± 42	62 ± 40	74 ± 35	
Vitality	Intervention	61 ± 22	59 ± 19	60 ± 19	0.56
	Control	59 ± 17	57 ± 16	60 ± 19	
General health	Intervention	62 ± 19	64 ± 17	63 ± 20	0.69
	Control	63 ± 17	71 ± 18	65 ± 21	
Social functioning	Intervention	71 ± 23	74 ± 20	79 ± 22	0.84
	Control	70 ± 23	79 ± 20	78 ± 24	
Role-emotional	Intervention	50 ± 44	64 ± 43	74 ± 39	0.55
	Control	58 ± 40	71 ± 39	77 ± 38	
Mental health	Intervention	64 ± 17	77 ± 15	75 ± 15	0.61
	Control	67 ± 14	73 ± 15	73 ± 17	
Pain	Intervention	67 ± 31	76 ± 21	77 ± 26	0.97
	Control	70 ± 23	76 ± 18	77 ± 21	
Quality of life* VAS (0–100) (N = 81)	Intervention	62 ± 20	73 ± 16	69 ± 23	0.65
	Control	61 ± 21	70 ± 17	67 ± 22	
Overall work ability* (WAI) (0–10) (N = 78)	Intervention	5.5 ± 3.0	6.6 ± 2.0	6.7 ± 2.7	0.92
	Control	5.5 ± 3.2	6.8 ± 1.9	7.0 ± 2.4	
Overall work limitations^*^ (WLQ) (0–100) (N = 71)	Intervention	Na	28 ± 16	26 ± 17	0.64
	Control	Na	25 ± 15	21 ± 15	

Mean ± standard deviation; *Higher scores represent a higher level of functioning/well-being/quality of life, work ability, and lower levels of work functioning. **P-value represents the interaction effect of time and group

### Secondary Outcomes—Work Ability, Work Functioning

Work ability improved statistically significant over time most prominently from baseline to 1 year of follow-up and remained stable from 1 year of follow-up to 2 years of follow-up and did not differ between groups (Table 2). Work functioning improved statistically significant over time (p = 0.001) and did not differ between groups (Table 2).

### Predictors of Time to RTW

Factors predicting a longer time to RTW in the univariate Cox regression analyses were (Table 3): having received chemotherapy (HR = 2.431 [95% Confidence Interval (CI) 1.588–3.726] p < 0.001), a lower level of education (low vs. medium/high) (HR = 1.65, 95% CI 1.076–2.52 p = 0.022) and a lower overall work ability (HR = 1.088 [95% Confidence Interval (CI) 1.04–1.168] p = 0.018).

**Table 3:** Factors predicting a longer time to RTW in the univariate Cox regression analyses

	Hazard Ratio (HR) [95% Confidence Interval]	p-Value
Having received chemotherapy (yes vs. no)	2.431 [1.588–3.726]	p < 0.001
Level of education (low vs. medium/high)	1.65 [1.076–2.52]	p = 0.022
Overall work ability	1.088 [1.04–1.168]	p = 0.018

## Discussion

The aims of our study were to study effectiveness of the hospital-based work support intervention for cancer patients at 2 years of follow-up and to identify which early factors predict time to RTW at 2 years of follow-up. We found high RTW rates and large variation in time to RTW between patients. We found no differences between the intervention group and the control group on time to RTW or the RTW rate nor on any of the secondary outcomes. In addition, we found that those who received chemotherapy, have a low level of education, and a lower overall work ability are at risk of longer time to RTW.

### Strengths and Weakness

Due to the inclusion of a small sample size according to our power analysis [15] and a selective lost-to-follow-up of 21% we were only able to include 106 patients in the final analyses. This led to higher uncertainty and bias in the results. However, as we found that a substantial part of the cancer patients had a work change in their work status (e.g., people returning to work at 1 year follow-up are on recurrent sick-leave at 2 years of follow-up), we are of the opinion that our results are of added value to the literature. Finally, our study was limited to breast and gynaecological cancer patients. As gender as well as treatment type may influence time to RTW and RTW rate, our results are not generalizable to other cancer types.

A strength of our study includes the use of a low-cost intervention that was easily accepted as part of psycho-oncological cancer care that could be carried out in different hospitals without much deviation from usual cancer care [19].

### Interpretation of Findings

Several explanations can be sought for the similar outcomes between groups and thereby also the similar results between 1 and 2 years of follow-up. This includes insufficient training of the nurses to deliver education and support, lack of involvement of the occupational physician and employer in the intervention, and weaknesses in the study design such as contamination between groups as nurses delivered to both groups psycho-oncological care [18]. Progress of insight into our results provides us to elaborate on two possible explanations in greater detail, i.e. selection bias and operationalisation of the primary outcome RTW.

As 6 of the 11 hospitals that we contacted participated in our study [19] and as we found high return-to-work rates compared to large population based studies on breast cancer survivors (71% versus 87%) [33], we conclude that a selection of hospitals and patients have participated in our study. Hospitals that were motivated to deliver the work support intervention as part of psycho-oncological care were willing to participate. This led to less contrast between the control group and the intervention group as nurses were very keen on delivering work-related support to patients in de control group as well. Besides, a selection of patients that participate in research is a widely reported problem, which has increased over the past years [34]. In general, persons who have completed a higher level of education, are older, married and are native tend to be overrepresented in research [34]. In our type of research, this is even more problematic as a lower level of education and lower socio-economic stats (SES) is a predictor of unemployment [35] and being younger is a predictor of unemployment in breast cancer survivors [36]. It is thus of the utmost importance in this type of research that one has either a representative sample in their study or a selective population that has a high risk of unemployment. For our results this means that we cannot be certain whether the intervention is not effective or that the selective participation of hospitals and patients led to no differences between groups.

A solution to this problem could be to develop a measurement instrument that identifies the group at risk of unemployment. Thereafter, we could give special attention to the development and delivery of the intervention to the characteristics of the high-risk group. This strategy has for example been applied in the screening method fitting with the multi-disciplinary cardiac rehabilitation guideline as this guideline includes screening for needs regarding RTW [37]. The intervention content of this guideline depends on the outcome of the screening as for example patients who completed a lower level of education receive a more intense intervention [37]. Besides, from eHealth interventions it is known that dropout attrition rates are higher among patients who completed lower and middle level of education but that these patients evaluated the eHealth intervention better [38]. These findings make one aware that a one-size-fits-all approach does not suffice. In our opinion, when designing work support interventions, we should either, when targeting the whole population be able to modify certain features of the intervention to subgroups (e.g., based on level of education) or when targeting the high-risk group only, design the work support intervention in such a way that it matches the characteristics and needs of the high-risk group. Both strategies might decrease drop-out, maximize exposure to the intervention, increase satisfaction, and ultimately effectiveness.

The second possible explanation of our findings of high RTW rates and no differences between groups is the operationalising of the primary outcome RTW. The main idea of the intervention was to address RTW in an early stage, to alter misconceptions, improve communication with the occupational physician and involve the employer. However, whether or not someone is able to achieve the primary outcome RTW does not only depend on the intervention components as participants are also exposed to general labour market principles such as job loss due to the economic crisis. Therefore, a shift from assessing RTW towards assessing work-related goals as suggested by Wells et al. [39] or quality of working life [40] fits the intervention goals more closely. Operationalising RTW in such a manner, represents more reliable whether or not the intervention is considered effective or not. An appurtenant advantage of operationalising RTW in such a manner is the fact that the perception of the patients are taken into account. This might not be the case with the outcome ‘numbers of days from first day of sick-leave to full RTW’ as previous research found a shift in what aspects of their work, was considered important after a diagnosis of cancer [39]. Thus resulting in operationalising RTW that also represents the perspective of the target population [41].

In contrast to the analysis of our results after 1 year of follow-up we now found an inconsistency between the two definitions of the primary outcome ‘RTW’ in six participants. These participants had returned to work for at least 4 weeks consecutively but were not working anymore at 2 years of follow-up. This was the case when someone for instance lost her job after full RTW. In the Kaplan-Meijer survival analysis, these participants are considered ‘RTW’ but when analysing the RTW rate these participants are considered ‘*not* RTW’. As a consequence, an intervention with the same outcome, might be effective in increasing the time to RTW but not in increasing the RTW rate at follow-up or vice versa. For that reason, it is important for further research to consider which operationalisation of the primary outcome RTW is considered most relevant for that type of work support intervention to be able to interpret the outcome better.

In accordance with previous research, we found that low work ability, receiving chemotherapy and low level of education were predictors of longer time to RTW [9, 36]. However, in contrast with previous research we found that fatigue and several work-related variables were not predictors to time until RTW [35]. Differences between our findings and previous research could be explained by the fact that potential predictors were assessed at different time points while it is known that this influence whether or not a variable is a predictor. When developing prediction models it is therefore important to keep in mind that the predictor is assessed at the same time point as the prediction model is intended to be used in practice, to guarantee reliability of the prediction model.

In conclusion, as we found high return-to-work rates compared to national register-based studies and as we found no differences between groups, future studies should focus on reaching the group at risk. The group at risk may consist of patients who receive chemotherapy, have a low level of education and have a low work ability at diagnosis.
